# A Rare Case of a Large Sinonasal Neurofibroma

**DOI:** 10.7759/cureus.32202

**Published:** 2022-12-05

**Authors:** Antonios Skalias, Paraskevi Karamitsou, Alexandros Poutoglidis, James Philip Skliris, Spyridon Gougousis

**Affiliations:** 1 Department of Otorhinolaryngology-Head and Neck Surgery, “G. Papanikolaou” General Hospital, Thessaloniki, GRC; 2 Department of Otorhinolaryngology-Head and Neck Surgery, "G. Papanikolaou” General Hospital, Thessaloniki, GRC; 3 Department of Pathology, “G. Papanikolaou” General Hospital, Thessaloniki, GRC

**Keywords:** sinonasal tract, sinonasal tumor, nose, endoscopic sinus surgery, peripheral nerve sheath tumor, neurofibroma

## Abstract

A neurofibroma is a benign peripheral nerve sheath tumor. Its appearance in the nose and paranasal sinuses is extremely rare. We present the case of a 61-year-old female with a large sinonasal neurofibroma. The patient was referred to our department due to the findings of a large invasive lesion originating from the left sinus with extension to the adjacent structures on computed tomography. A thorough examination revealed a mass within the left nasal cavity and exophthalmos. The initial symptoms of the disease probably appeared three years ago when she reported that she developed facial swelling following dental work. In the following period and due to reported blurred vision, she consulted with several medical specialists without receiving a diagnosis, while later she visited an otorhinolaryngologist, complaining of ear fullness, and local treatment was prescribed. Due to persistent symptoms, the patient was finally referred for computed tomography. Upon arrival at our department, she underwent a biopsy, which revealed the existence of a neurofibroma. The patient underwent endoscopic resection of the tumor and remains under close follow-up with no signs of recurrence. Sinonasal neurofibroma is a rare condition that presents with non-specific symptoms and may take years to reach a diagnosis. Open or endoscopic surgical resection seems to offer satisfactory results; however, similar cases reported in the literature are scarce.

## Introduction

Neurofibroma is a benign peripheral nerve sheath tumor (PNST) composed of mixed Schwann cells, perineural-like cells, and intraneural fibroblasts [1]. It can occur either as multiple lesions in individuals affected by neurofibromatosis type 1 (NF1) or as a solitary neurofibroma [2]. Approximately 25% to 45% of neurofibromas arise in the head and neck region; however, only 4% involve the nasal cavity and paranasal sinuses [3]. Due to its slow growth and the non-specific nature of the symptoms, it often takes more than four years to reach a diagnosis [4]. Only a few cases have been reported in the current literature [4-12]. We present the case of a patient with a sinonasal neurofibroma of exceptional size treated in our department.

## Case presentation

**Figure 1:**
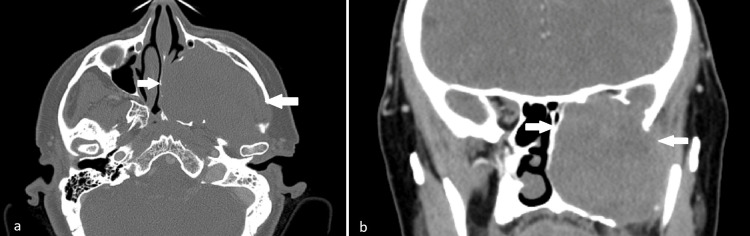
Computed tomography imaging reveals a large sinonasal mass. (a) Axial view; (b) Coronal view

**Figure 2:**
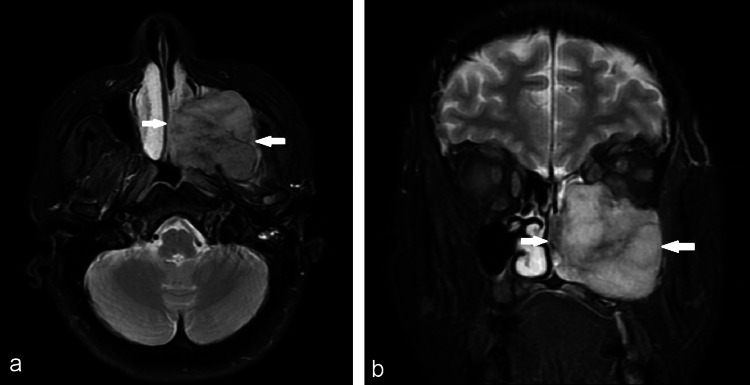
Magnetic resonance imaging of the same patient (T2 sequence). (a) Axial view; (b) Coronal view (a) axial view; (b) coronal view

A 61-year-old female presented to our department due to worrisome findings in her computed tomography (CT) scan, including a large mass involving the left nasal cavity and ipsilateral sinuses and spreading to the left orbit and cranial fossa with bony destruction (Figures 1a, 1b). Clinically, we observed a large mass occupying the left nasal cavity and ipsilateral exophthalmos. Magnetic resonance imaging (MRI) was requested to better estimate the extent of the lesion (Figures 2a, 2b).

The patient worked as a house cleaner and had no pertinent medical history. The initial symptoms may have appeared three years ago when, following a molar extraction, she noticed ipsilateral facial swelling, including her eyelids. She visited several physicians, including an otorhinolaryngologist, a neurologist, an ophthalmologist, and a dentist, none of whom presented her with a diagnosis. Meanwhile, she noticed her vision blurring. Almost two years later, she visited an otorhinolaryngologist due to complaints of ear fullness and was treated with antibiotics and nasal decongestants. Finally, a few months later, she was referred for a CT by a general practitioner and later presented to our department.

**Figure 3:**
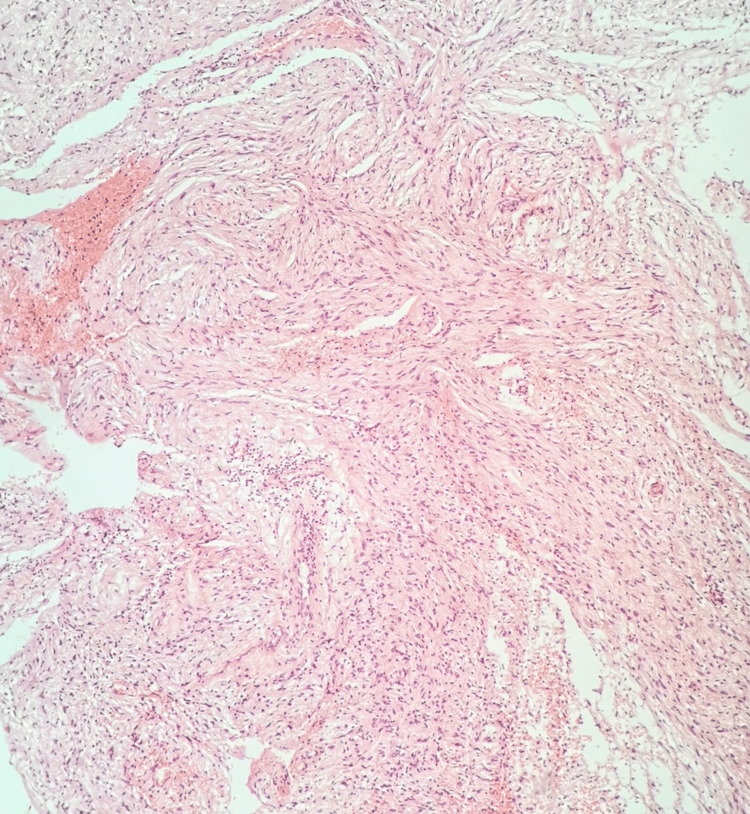
Neoplastic cells with bland, spindle-shaped nuclei and hazy borders organized in low-density bundles. Hematoxylin and eosin, 10X.

**Figure 4:**
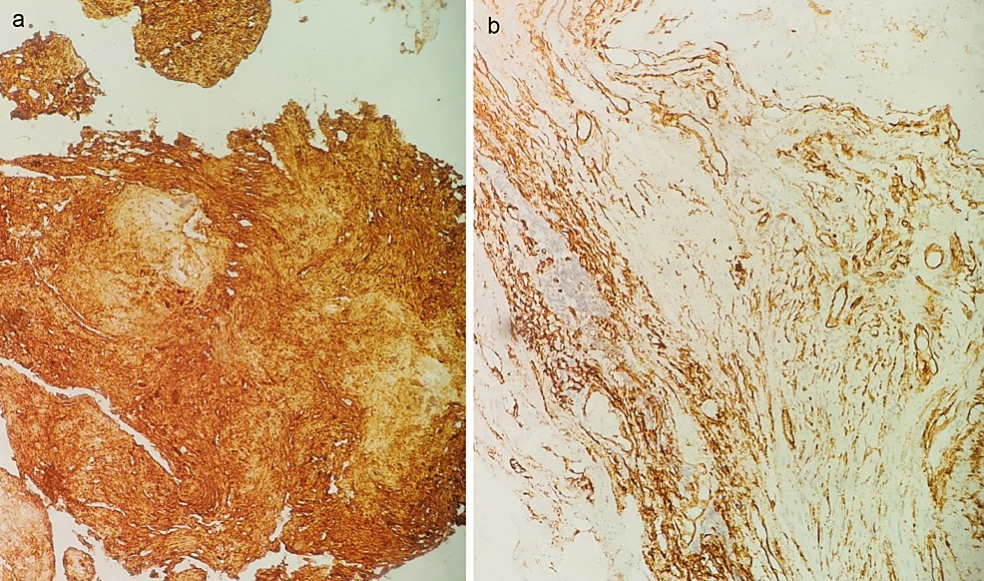
Immunohistochemistry, 20X. (a) S-100 positivity is exhibited. (b) Focal CD34 positivity is also evident.

A biopsy via a transnasal endoscopic approach was scheduled. Histopathological and immunohistochemical examination demonstrated findings indicative of neurofibroma (Figures 3, 4a, 4b). On further investigation, the patient had no findings raising suspicion for neurofibromatosis type 1 (NF1), such as café-au-lait spots, Lisch nodules, or first-degree relatives with NF1. She denied any other possible approaches and only consented to undergo endoscopic sinus surgery (ESS). The surgery was performed under general anesthesia, with the aim of resecting as much of the lesion as possible. The histopathological examination confirmed the initial diagnosis. The patient remains under close and meticulous monitoring, without signs of tumor recurrence.

## Discussion

Neoplasms of Schwann cell origin occur only rarely in the nasal cavity and paranasal sinuses [13]. These can include neurilemomas (schwannomas), malignant schwannomas, or less frequently, neurofibromas [14]. Both solitary and NF1-associated neurofibromas are exceptionally rare in the sinonasal tract.

A series of such cases revealed that there was no gender predilection among patients. The mean age was 46.2 years (ranging from 26 to 75 years), with a mean duration of symptoms of 42.9 months. The symptoms were non-specific and included a mass lesion along with obstruction, pain, and epistaxis. The tumor size ranged from 0.4 to 4.1 cm, which was significantly smaller than our case. The lesions were unilateral, most commonly affecting the nasal cavity, followed by the maxillary sinus, or more than one site [4].

On pathologic examination, neurofibromas are identified below an intact mucosa with no encapsulation. The lesional cells are arranged in irregular interlacing fascicles, bundles, or single spindled cells, separated by variable proportions of coarse collagen bundles, which give the characteristic "shredded carrot" appearance. A myxoid to loose connective tissue stroma is seen in most cases [4,13].

Immunohistochemical findings include positivity for S100 protein and glial fibrillary acidic protein (GFAP), Sry-related HMg-Box gene 10 (SOX10), neurofilament protein (NFP), and calretinin, which highlight the axons, and CD34, which often highlights nerve twigs and pseudomeissnerian corpuscles. In the setting of NF1, features indicative of a malignant change in neurofibroma are increased cellularity, diffuse atypia, and mitotic activity [4,14].

The nasal cavity may host a variety of rare neoplasms [15]. Misdiagnosis of neurofibromas is not uncommon. Differential diagnosis of schwannomas is extremely difficult based on symptoms, endoscopy, and imaging alone and should be based on histopathological and immunohistochemical studies [16]. Other lesions that are confused with sinonasal tract neurofibromas are dermatofibrosarcoma protuberans, fibrosarcoma, meningioma, leiomyoma, solitary fibrous tumor, leiomyosarcoma, malignant fibrous histiocytoma, low-grade sinonasal sarcoma with neural and myogenic features, proliferative fasciitis, "inflammatory pseudotumor," fibromatosis, and fibrous histiocytoma [4]. Additionally, the physician should remain vigilant for NF1 signs and symptoms such as café-au-lait spots, more than one neurofibroma, Lisch nodules, and first-degree relatives with NF1 [17].

Surgical excision of sinonasal tumors significantly affects the patient's quality of life [18]. Sinonasal neurofibroma treatment varies from an endoscopic approach [5,6,9,10] to external approaches (e.g., midfacial degloving, lateral rhinotomies) [7, 8, 11]. In most cases, there was no recurrence at follow-up.

## Conclusions

Neurofibroma of the sinonasal tract is an extremely rare lesion, with only a few cases reported in the current literature. The non-specific nature of its symptoms, as well as the low clinical suspicion due to its rarity, often lead to a delayed or erroneous diagnosis. Both endoscopic and open approaches seem to offer satisfying results. However, as more cases are discovered and reported, physicians will become more aware of this rare clinical entity, and treatment options will be better investigated and understood.
